# Outbreak of IncX8 Plasmid–Mediated KPC-3–Producing Enterobacterales Infection, China

**DOI:** 10.3201/eid2807.212181

**Published:** 2022-07

**Authors:** Lan Chen, Wenxiu Ai, Ying Zhou, Chunyang Wu, Yinjuan Guo, Xiaocui Wu, Bingjie Wang, Lulin Rao, Yanlei Xu, Jiao Zhang, Liang Chen, Fangyou Yu

**Affiliations:** Ningbo First Hospital, Ningbo, China (L. Chen);; First Affiliated Hospital of Wenzhou Medical University, Wenzhou, China (W. Ai, C. Wu, L. Rao, J. Zhang, F. Yu);; Shanghai Pulmonary Hospital, Tongji University School of Medicine, Shanghai, China (Y. Zhou, Y. Guo, X. Wu, B. Wang, Y. Xu, F. Yu);; Hackensack Meridian Health Center for Discovery and Innovation, Nutley, New Jersey, USA (L. Chen);; Hackensack Meridian School of Medicine, Nutley (L. Chen)

**Keywords:** Enterobacterales, Klebsiella pneumoniae, KPC-3, carbapenemase, transmission, horizontal transfer, plasmid, outbreak, bacterial infections, bacteria, antimicrobial resistance, China

## Abstract

Carbapenem-resistant Enterobacterales (CRE) infection is highly endemic in China; *Klebsiella pneumoniae* carbapenemase (KPC) 2–producing CRE is the most common, whereas KPC-3–producing CRE is rare. We report an outbreak of KPC-3–producing Enterobacterales infection in China. During August 2020–June 2021, 25 *bla*_KPC-3_–positive Enterobacteriale isolates were detected from 24 patients in China. Whole-genome sequencing analysis revealed that the *bla*_KPC-3_ genes were harbored by IncX8 plasmids. The outbreak involved clonal expansion of KPC-3–producing *Serratia marcescens* and transmission of *bla*_KPC-3_ plasmids across different species. The *bla*_KPC-3_ plasmids demonstrated high conjugation frequencies (10^−3^ to 10^−4^). A *Galleria mellonella* infection model showed that 2 sequence type 65 K2 *K. pneumoniae* strains containing *bla*_KPC-3_ plasmids were highly virulent. A ceftazidime/avibactam in vitro selection assay indicated that the KPC-3–producing strains can readily develop resistance. The spread of *bla*_KPC-3_–harboring IncX8 plasmids and these KPC-3 strains should be closely monitored in China and globally.

Carbapenemase-producing Enterobacterales (CPE) have emerged as important nosocomial pathogens and are a global public health concern because of the high prevalence of CRE infection and its associated mortality rate. Currently, *Klebsiella pneumoniae* carbapenemase (KPC) is the most clinically important carbapenemase globally (*1*,*2*). Since its first discovery in 1996 (*3*), more than 90 KPC variants have been documented, of which KPC-2 and KPC-3 are the most common clinical variants (*4*). *bla*_KPC_ genes are frequently harbored by Tn*4401* or non-Tn*4401* mobile elements (NTM_KPC_) (*5*), and the spread of *bla*_KPC_ has been primarily associated with transmissible plasmids, belonging to different incompatibility groups (e.g., IncFII, IncI2, IncX, IncA/C, IncR, IncN, and ColE) (*5*).

China is regarded as a CRE-endemic region where *K. pneumoniae*, *Escherichia coli,* and *Enterobacter cloacae* complex are the most common CRE species (*6*,*7*). Among the CREs, ≈80%–90% were carbapenemase-producers, including >90% carbapenem-resistant *K. pneumoniae* and *E. coli* and ≈80% of carbapenem-resistant *E. cloacae* strains. For carbapenemase genes, the *bla*_KPC-2_ was the most dominant type (≈60%), followed by *bla*_NDMs_ and *bla*_IMPs_. Interestingly, other KPC variants, especially KPC-3, are rarely detected in China. Compared with KPC-2, KPC-3 differs by a single amino acid substitution (H272Y) and shows higher hydrolysis efficiency against oxyimino-cephalosporins and carbapenems (*8*). In most KPC-endemic regions, including United States and countries in Europe, KPC-3 showed similar prevalence as that of KPC-2 enzyme, and both KPC variants were frequently detected in clinical CRE isolates. Despite China being KPC-endemic, KPC-3 has only been sporadically reported in China (*9*–*13*).

In this study, we describe a hospital outbreak of KPC-3–producing *Enterobacterales* involved with multiple species, including *Serratia marcescens*, *K. pneumoniae*, *Escherichia coli*, *Enterobacter hormaechei*, and *Proteus mirabilis* in mainland China. We obtained approval for the study from Ningbo First Hospital Ethics Committee (approval no. 2021RS095).

## Materials and Methods

During August 1, 2020–June 30, 2021, we collected from a tertiary hospital in Ningbo, Zhejiang Province, China, 25 nonrepeated KPC-3–producing Enterobacterales isolates showing reduced susceptibility to carbapenems. None of the patients from whom the isolates were taken had international travel history in the preceding 3 months. We detected the presence of carbapenemase genes, including *bla*_KPC_, *bla*_NDM_, *bla*_OXA-48–like_, *bla*_VIM_, and *bla*_IMP_, by using PCR, followed by Sanger sequencing (*14*,*15*). We initially determined speciation by using matrix-assisted laser desorption/ionization time-of-flight mass spectrometry and analyzed results by using the Vitek MS database (bioMérieux, https://www.biomerieux.com); we later confirmed the results by using whole-genome sequencing (WGS) analysis.

We performed antimicrobial susceptibility testing and modified carbapenem inactivation and applied WGS to explore the molecular features of the isolates (Appendix 1). We also conducted conjugation and electroporation experiments (and performed pulsed-field gel electrophoresis (PFGE) and S1-nuclease PFGE (Appendix 1). We applied string test to examine the Hypermucoviscous phenotypes of *K. pneumoniae* strains and *Galleria mellonella* infection model to evaluate the virulence potential of sequence type (ST) 65 K2 *K. pneumoniae* strains (Appendix 1). We applied a ceftazidime/avibactam in vitro selection assay to evaluate whether the KPC-3-producing strains were easily selected to be resistant to ceftazidime/avibactam (Appendix 1). 

We submitted the complete nucleotide sequences of the plasmids pFK3112-KPC-3 and pCG2111-KPC-3 to GenBank (accession nos. CP081509 [pFK3112-KPC-3] and CP081510 [pCG2111-KPC-3]). We also deposited the raw reads of the genomes we sequenced in GenBank (Bioproject accession no. PRJNA354234).

## Results

### Outbreak Description of *bla*_KPC-3_–harboring *Enterobacterales*

During August 1, 2020–June 30, 2021, we detected 25 KPC-3–producing Enterobacterales isolates in patients of a tertiary hospital in eastern China, including 18 *Serratia marcescens*, 3 *K. pneumoniae*, 1 *E. coli*, 2 *E. hormaechei*, and 1 *Proteus mirabilis*. The 25 isolates were from 24 patients; 1 patient had 2 isolates from sputum (FK3015) and blood (FK3018). The strains were recovered from sputum (n = 19), blood (n = 3), urine (n = 1), puncture fluid (n = 1), and bile (n = 1).

The first KPC-3 strain (*S. marcescens* CG2008) was isolated in August 2020, and the patient was admitted to the intensive care unit (ICU) 1 (building 3) with unconsciousness attributable to a head injury sustained in an accident. After 20 days of the patient’s hospitalization, we detected a carbapenem-resistant *S. marcescens* in the patient’s sputum. After that, we detected 4 additional carbapenem-resistant *S. marcescens* strains in the same ICU ward during August 2020–May 2021 (Appendix 2 Table 1). Starting in January 2021, we also found carbapenem-resistant *S. marcescens* strains (n = 6) in another ICU (building 2, ICU-2) and the wards of cardiology (building 2) (n = 2), emergency (building 3) (n = 1), and hepatobiliary and pancreatic surgery (building 3) (n = 1). In addition, starting in September 2020, we identified these KPC-3–producing strains in other *Enterobacterales* species in ICU-2 (*K. pneumoniae*), the coronary heart disease care unit (*K. pneumoniae*), ICU-1 (*P. mirabilis* and *E. coli*), the infectious disease ward (building 6) (*E. hormaechei*), and the hepatobiliary and pancreatic surgery ward (*E. hormaechei*) (Appendix 2 Table 1).

**Figure 1:**
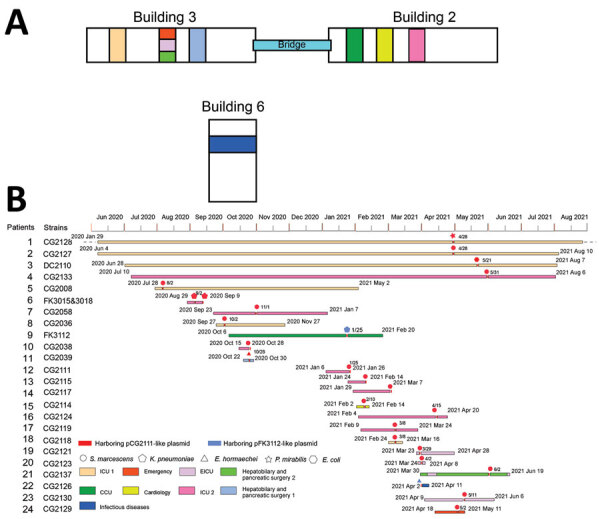
Characteristics of outbreak of *Klebsiella pneumoniae* carbapenemase 3–producing *Enterobacterales* infection at a tertiary hospital in Ningbo, Zhejiang Province, China, August 1, 2020–June 30, 2021. A) Spatial location features of the hospital. B) Timeline of events during the outbreak. CCU, cardiac care unit; EICU, emergency intensive care unit; ICU, intensive care unit.

Of the patients, 23/24 were admitted into wards in medical buildings 2 and 3, including the 2 ICUs, in our hospital (Figure 1, panel A); 18 of them were infected with the *S. marcescens* (*bla*_KPC-3_). Most patients shared the same ward during the same time, especially the patients from ICU-1 and ICU-2. The 2 buildings were connected by a pedestrian bridge, and frequent movement of persons (medical workers, patients, and visitors) and portable medical devices occurred between the 2 buildings, providing many opportunities for the intrahospital transmission of bacterial pathogens between buildings and wards.

Average age of these patients was 72 years (range 39–94 years), and most (79%) were men. All but 1 KPC-3 isolates were detected >2 days after admission (range 4–328 days). KPC-3 *E. coli* isolate CG2126 (from patient 22) was detected from the blood sample of a patient on the same day of admission in April 2021; however, this patient had cholangiocarcinoma and had been hospitalized in the hepatobiliary and pancreatic surgery ward 2 weeks earlier. Most patients had serious underlying diseases, including type 2 diabetes mellitus (n = 8), hypertension (n = 4), hypoproteinemia (n = 4), and cerebral infarction (n = 2). Most patients had received β-lactam antimicrobial treatments, such as piperacillin/tazobactam (n = 17), meropenem (n = 12), tigecycline (n = 12), and cefoperazone/sulbactam (n = 9). Most patients (95.8%) underwent invasive procedures that involved medical devices, including attachment of a ventilator (23/24), deep vein intubation (9/24), and attachment of a urinary catheter (4/24) (Appendix 2 Table 1). In addition, most patients had prolonged hospital stays (range 8–462 days), including 6 patients who were hospitalized for >6 months (Figure 1, panel B). The prognosis of some patients was poor. Half of the patients (n = 12) had deteriorating health conditions during discharge, and 3 patients died during their hospital stay (Appendix 2 Table 1).

Starting in mid-2021 (and coinciding with the COVID-19 epidemic), the hospital enacted enhanced infection control measures, including chlorhexidine skin cleaning for ICU patients, improved hand hygiene compliance in healthcare workers, easy access to hand-hygiene supplies, restriction of hospital visitors, decontamination of the patients’ environment, and enhanced disinfection of medical equipment. Those measures also help to control the KPC-3 CRE outbreak, and only 1 carbapenem-resistant *S. marcescens* (attributable to NDM) was detected from August 2021 (data not shown).

### Antimicrobial Susceptibility and Carbapenem Inactivation Assay

We then examined the susceptibility of the 25 isolates against 18 antibiotics (Appendix 2 Table 2). Our results indicated these isolates were all multidrug-resistant, exhibiting high-level resistance to all β-lactam antibiotics, including carbapenems, but remained susceptible to amikacin, gentamicin, and ceftazidime/avibactam (except CG2126). The *E. hormaechei* isolate CG2126 was resistant to ceftazidime/avibactam because of the co-existence of *bla*_NDM-1_ (Appendix 2 Table 1). Modified carbapenem inactivation method results confirmed that all 25 isolates were carbapenemase producers, consistent with the presence of *bla*_KPC-3_ (or *bla*_NDM-1_) genes among these isolates.

### Genomic Phylogeny of KPC-3–producing Enterobacterales

**Figure 2:**
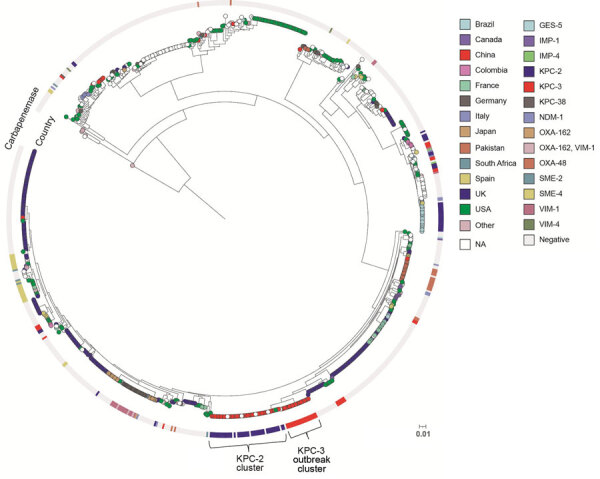
Core-genome phylogenetic tree of 748 *Serratia marcescens* genomes from the National Center for Biotechnology Information RefSeq database (https://www.ncbi.nlm.nih.gov/refseq) and 18 KPC 3–producing strains from an outbreak of KPC 3–producing Enterobacterales infection at a tertiary hospital in Ningbo, Zhejiang Province, China, August 1, 2020–June 30, 2021. The isolation country is color-coded and illustrated at the tips. Carbapenemases are presented as a color-coded outer circle. The tree was rooted in the midpoint. Scale bar represents 0.01 mutations per nucleotide position. KPC, *K. pneumoniae* carbapenemase; NA, not available; UK, United Kingdom; USA, United States.

We first conducted a core-genome phylogenetic analysis by using Parsnp (*16*) and compared our KPC-3–producing *S. marcescens* genomes with 748 *S. marcescens* genome assemblies from the National Center for Biotechnology Information RefSeq database (https://www.ncbi.nlm.nih.gov/refseq [accessed October 1, 2021]). A total of 73 strains were from China. The core-genome tree showed that the 18 KPC-3 *S. marcescens* strains formed a single cluster and were phylogenetically close to another cluster of 44 strains, which mostly harbored KPC-2 and were from China (named KPC-2 cluster) (Figure 2). Further core single-nucleotide polymorphism (SNP) distance analysis showed that the 18 outbreak strains differed by an average of 7 core SNPs (range 0–18), indicating clonal expansion. They differed from the China KPC-2 cluster strains by an average of 7,400 core SNPs (range 7,388–7,428) and differed from the remaining strains from China by an average of 45,082 core SNPs (range 11,173–58,247), suggesting that the KPC-3 strains belonged to a unique clone, which is consistent with the core-genome phylogeny (Figure 2).

Three *K. pneumoniae* strains carried *bla*_KPC-3_, and they belonged to 2 STs (ST65 and ST967), containing KL2 and KL18 type capsules. The 2 K2 ST65 strains were isolated from the same patient. ST65 K2 strains belonged to prototypical hypervirulent *K. pneumoniae* clone, harboring a battery of virulence genes, encoding yersiniabactin (*ybt*), colibactin (*clb*), aerobactin (*iuc*), and Salmochelin (*iro*). The 2 K2 strains also contained an IncHIB-FIB virulence plasmid, harboring the regulator of mucoid phenotype A genes *rmpA* and *rmpA2*. The 2 ST65 strains only differed by 2 core SNPs. The *E. hormaechei* strain CG2126 belonged ST127, and the *E. coli* strain was from a novel ST.

### *bla*_KPC-3_–harboring IncX8 Plasmids

Two representative *bla*_KPC-3_-plasmids (pCG2111-KPC-3 and pFK3112-KPC-3) were completely sequenced. De novo assembly of the plasmid sequences generated a single head-to-tail contig for each plasmid. PlasmidFinder 2.1 assigned the 2 plasmids as the IncX5_2 (GenBank accession no. MF062700), whereas a recent study has reassigned IncX5_2 plasmids as a novel IncX8 group (*17*).

**Figure 3:**
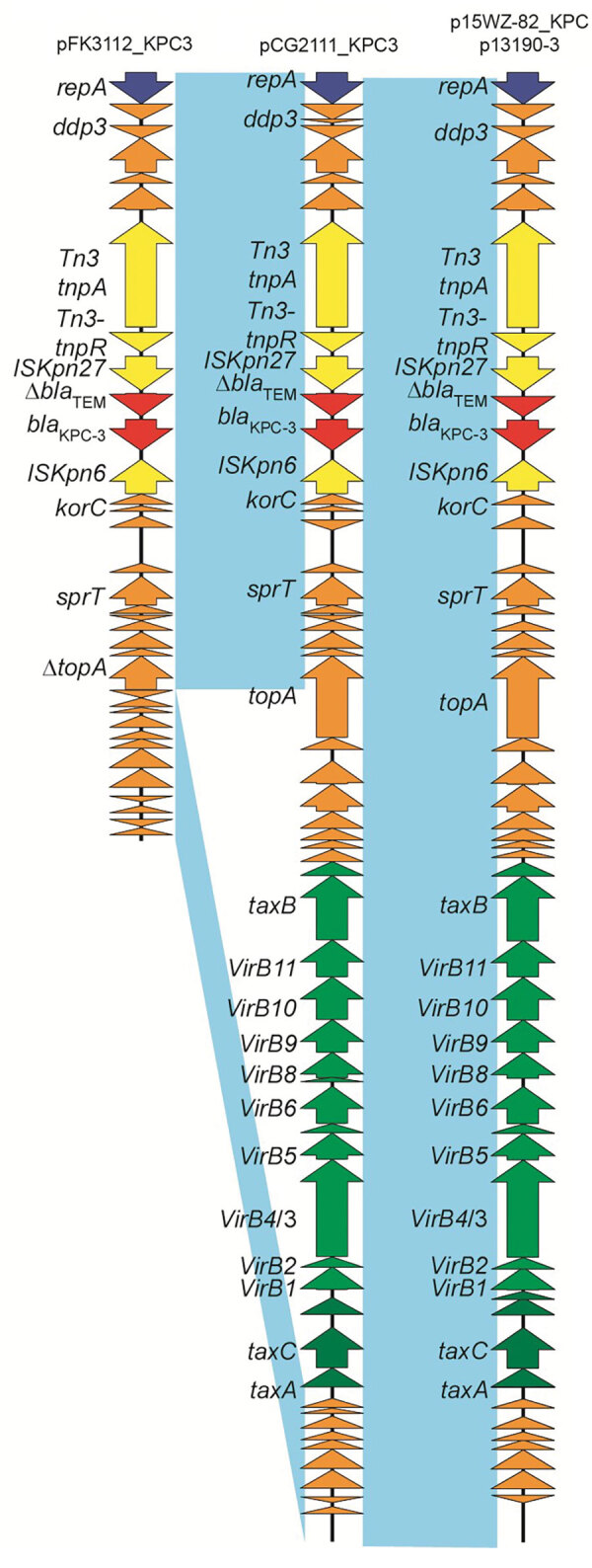
Comparative analysis of the *bla*_KPC-3_–harboring plasmid pCG2111_KPC3 (GenBank accession no. CP081510), pFK3112_KPC3 (GenBank accession no. CP081509), p15WZ-82-KPC, and *Klebsiella pneumoniae* p13190 in isolates from an outbreak of KPC 3–producing Enterobacterales infection at a tertiary hospital in Ningbo, Zhejiang Province, China, August 1, 2020–June 30, 2021. Open reading frames are portrayed by arrows and are depicted in different colors on the basis of their predicted gene functions. Red arrows indicate resistance genes, and green arrows indicate genes associated with the type IV secretion system. Orange arrows represent the backbone genes of the plasmid, and yellow arrows denote the mobile elements. Light blue shading denotes shared regions of homology among different plasmids. KPC, *K. pneumoniae* carbapenemase.

**Figure 4:**
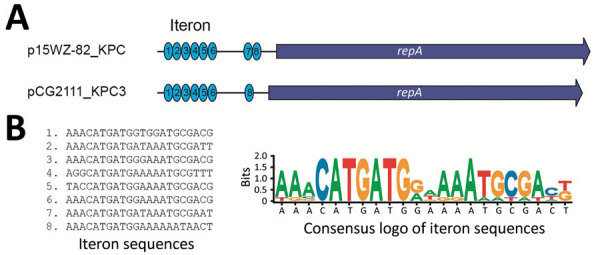
The iteron difference between pCG2111-KPC-3 and p15WZ-82_KPC. A) p15WZ-82_KPC IncX8 plasmids have eight 22-bp iteron copies located upstream from the replication gene, whereas pCG2111-KPC-3 only has 7 copies of iteron and the seventh iteron (in comparison to p15WZ-82_KPC) was deleted. B) The sequences of the 8 iterons are listed and a SeqLog (https://pypi.org/project/seqlog) presentation of the conserved motif is shown. KPC, *K. pneumoniae* carbapenemase.

The pCG2111-KPC-3 was 41,852 bp in length, with an average G+C content of 46%, and harbored 57 predicted open reading frames, with *bla*_KPC-3_ the only intact antimicrobial resistance gene. A BLAST search (https://blast.ncbi.nlm.nih.gov/Blast.cgi) showed that pCG2111-KPC-3 was almost identical to plasmids p13190–3 (*bla*_KPC-2_–harboring; GenBank accession no. MF344555) (*17*), isolated from ST392 *K. pneumoniae* in 2013, and p15WZ-82_KPC (*bla*_KPC-2_–harboring; GenBank accession no. CP032355) (*18*), isolated from ST595 *K. variicola* in 2015 in China. In addition, both *bla*_KPC-3_ (in pCG2111-KPC-3) and *bla*_KPC-2_ (in p13190–3 and p15WZ-82_KPC) genes were carried by a conserved Tn*3* transposon, with the structure of *tnpA*-*npR*-IS*kpn27*-Δ*bla*_TEM_-*bla*_KPC-2/3_-IS*kpn6*. We observed 2 major differences between pCG2111-KPC-3 and the other 2 *Klebsiella* IncX8 plasmids: first, pCG2111-KPC-3 harbors *bla*_KPC-3_, whereas the other 2 carry *bla*_KPC-2_; second, the 2 *Klebsiella* IncX8 plasmids have 8 22-bp iterons located upstream from the replication gene, whereas pCG2111-KPC-3 only has 7 copies of iteron, and 1 iteron (AAACATGATGATAAATGCGAAT) was deleted (Figure 3, 4).

The pFK3112-KPC-3 plasmid was smaller (21,888 bp in length) and had an average G+C content of 48%, carrying the same IncX8 replicon, and harbored 36 predicted open reading frames. In comparison to pCG2111-KPC-3, the only difference is that pFK3112-KPC-3 has a 19,964-bp deletion flanked by 5-bp repeat sequences of GCATC, encompassing the entire transfer operon from the type IA DNA topoisomerase gene *top* to the DNA distortion polypeptide gene *taxA* (Figure 3, 4).

We then used pCG2111-KPC-3 and pFK3112-KPC-3 as the reference sequences, and we used the reference mapping and mauve contig mover (*19*) functions in Geneious Prime 2020 (https://www.geneious.com) to reconstruct the IncX8 plasmids from the remaining 23 *bla*_KPC-3_–haroboring strains. The analysis showed that the *S. marcescens* (n = 18), *E. coli* (n = 1), *E. hormaechei* (n = 1) (CG2039), *P. mirabilis* (n = 1)*,* and ST65 K2 *K. pneumoniae* strains (n = 2) carried pCG2111-KPC-3–like plasmids. One *E. hormaechei* isolate (CG2126) had the pFK3112-KPC-3–like plasmid, with a ≈20-kb deletion in comparison to pCG2111-KPC-3.

**Figure 5:**
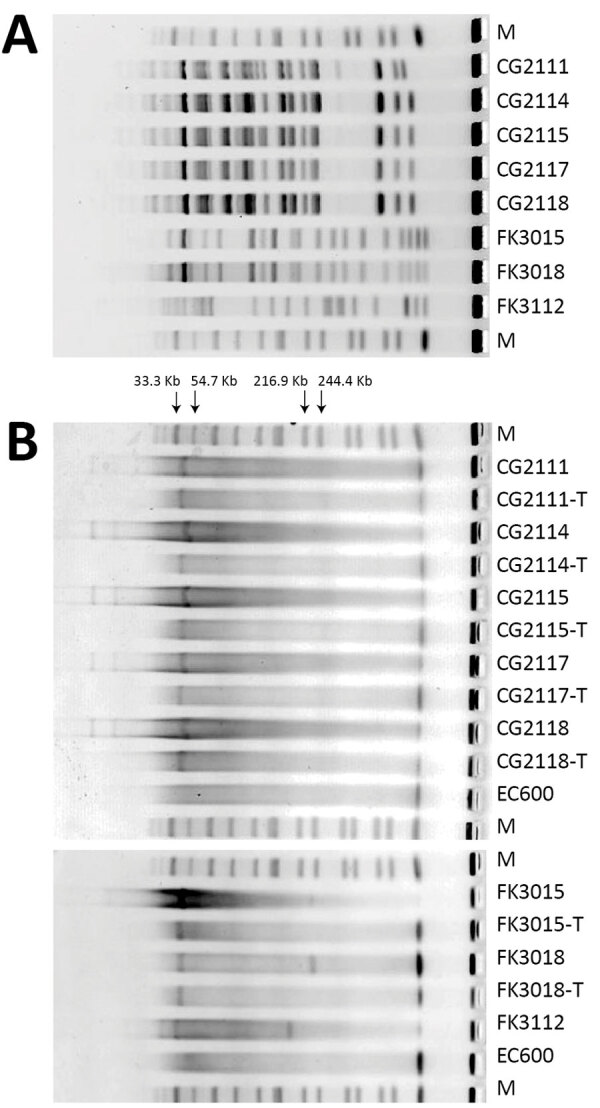
Pulsed-field gel electrophoresis (PFGE) profiles of selected *Klebsiella pneumoniae* carbapenemase 3–producing Enterobacterales strains isolated from patients at a tertiary hospital in Ningbo, Zhejiang Province, China, August 1, 2020–June 30, 2021. A) PFGE profiles. B) S1-nuclease PFGE profiles. EC, *Escherichia coli* EC; M, *Salmonella enterica* serotype Braenderup strain H9812; -T, the transconjugants of the corresponding strain.

We then used conjugation assay to evaluate the transconjugation ability and frequency of *bla*_KPC-3_–harboring IncX8 plasmids. We selected 7 strains, 5 *S. marcescens* and 2 ST65 K2 *K. pneumoniae*, as the donors and the *E. coli* EC600 as the recipient strain. The *bla*_KPC-3_ plasmids from these isolates were all successfully transferred to the recipient strain. The S1-nuclease PFGE pattern showed that all the 7 transconjugants had only 1 plasmid, at a size of ≈42 kb (Figure 5). The transfer frequencies of the 7 strains ranged from 1.57 × 10^−3^ to 7.8 × 10^−4^. The antimicrobial susceptibility testing results further confirmed the carbapenem resistance had been transferred to recipient strains. In addition, we tested the transconjugation ability of pFK3112-KPC-3 in *K. pneumoniae* FK3112, and the result showed that pFK3112-KPC-3 failed to conjugate, which is consistent with the sequence analysis showing the lack of *tra* operon.

### Ceftazidime/avibactam in vitro Selection Assays

Ceftazidime/avibactam has been increasingly used in China to treat CRE infections, especially those attributable to KPC producers (*20*,*21*). We conducted a subinhibitory concentration antimicrobial selection experiment in 24 KPC-3 strains (except CG2126) to examine their potential to develop ceftazidime/avibactam resistance. After induced selection by 1/2 MIC concentration of ceftazidime/avibactam, 22 isolates (all except 2 *S. marcescens*) developed resistance (MIC >16/4 μg/mL), and the resistant rate was as high as 91.7%. By contrast, we conducted the same in vitro selection in 24 *bla*_KPC-2_–harboirng *K. pneumoniae* strains. Only 1 strain developed ceftazidime/avibactam resistance (MIC >16/4 μg/mL) after 1/2 MIC induction, and the resistant rate was as low as 4.2%, which is significantly lower that of the induced resistance rate of KPC-3 strains (p<0.05). These results further suggested that these KPC-3-producing strains can be easily selected for resistance to ceftazidime/avibactam.

### *G. mellonella* Infection Model of KPC-3–Producing Hypervirulent K2 *K. pneumoniae* Strains

**Figure 6:**
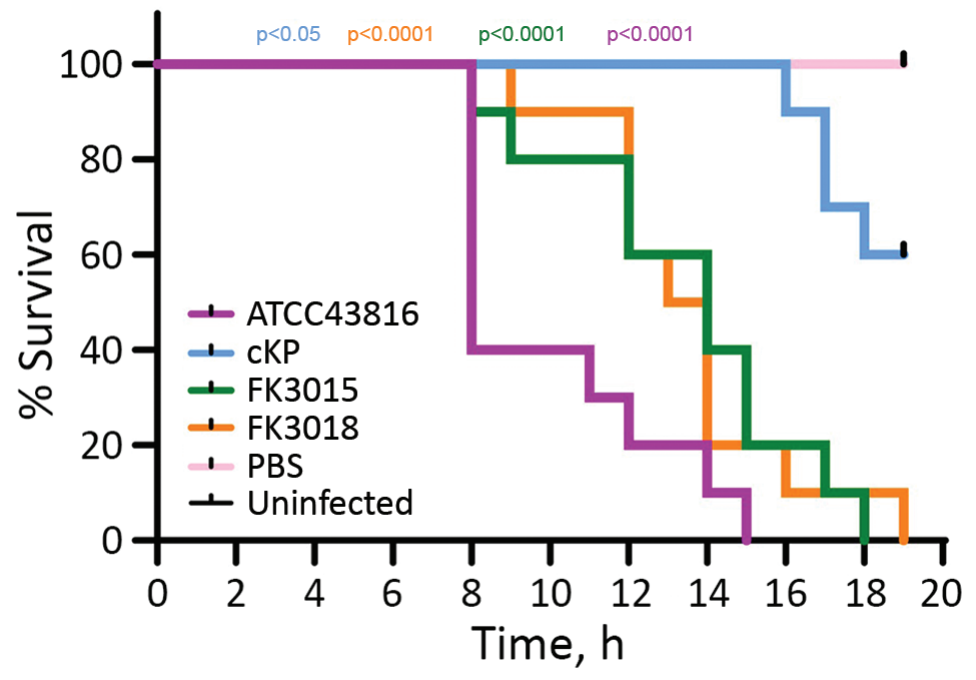
Survival of *Galleria mellonella* larvae infected with *Klebsiella pneumoniae* strains isolated from patients at a tertiary hospital in Ningbo, Zhejiang Province, China, August 1, 2020–June 30, 2021. A hypervirulent *K. pneumoniae* K2 strain ATCC 43816 was used as the positive control. A phosphate-buffered saline–injected and a pricking larval group (empty needle injection, uninfected) served as negative control groups. Data are pooled from >3 independent experiments with 10 larvae per group per run. The representative results are displayed. cKP, classical *K. pneumoniae*; PBS, phosphate-buffered saline.

*K. pneumoniae* K2 ST65 strains belong to the prototypical hypervirulent clone. A string test of the ST65 strains FK3015 and FK3018 showed positive results, consistent with their genotypes. To assess the potential virulence of these 2 isolates, we conducted the *G. mellonella* larvae infection experiment (Figure 6). After 20 hours of infection, *bla*_KPC-3_–harboring ST65 strains and the hypervirulent reference K2 *K. pneumoniae* strain ATCC 43816 showed a 100% mortality rate, which was significantly higher than that observed in larvae infected with nontoxic reference classical *K. pneumoniae* strains (p<0.05). These results indicate that the KPC-3 K2 ST65 *K. pneumoniae* are highly virulent and the acquisition of *bla*_KPC-3_–harboring IncX8 plasmids does not comprise the virulence potential in these strains.

## Discussion

In this study, we report a KPC-3–producing Enterobacterales outbreak in China. In China, the spread of *bla*_KPC-2_ was primarily associated with IncFII(pHN7A8)-R plasmids and with epidemic *K. pneumoniae* ST11 strains (*22*–*24*), whereas this KPC-3 outbreak was primarily associated with the clonal expansion of *S. marcescens* and was mediated by an uncommon IncX8 plasmid. We also detected horizontal transmission of *bla*_KPC-3_–harboring IncX8 plasmids in different Enterobacterales species. The *S. marcescens, E. coli*, *P. mirabilis*, *E. hormaechei* (CG2126), and the *K. pneumoniae* ST65 strains harbored the same plasmid as pCG2111-KPC-3, indicating horizontal transmission of *bla*_KPC-3_ plasmids in these strains. However, the origin of pCG2111-KPC-3 remains unclear. Although KPC-3 was initially detected in an *S. marcescens* isolate (CG2008), the possibility that CG2008 acquired this plasmid from other strains cannot be ruled out. *bla*_KPC-2_ IncX8 plasmids have been reported in *Klebsiella* isolates in China, and in our study 2 KPC-3–producing *K. pneumoniae* strains were recovered nearly at the same time as CG2008 (Appendix 2 Table 1). In addition, *S. marcescens* strains might have obtained pCG2111-KPC-3–like plasmids from other strains (e.g., *K. pneumoniae*), which was then followed by clonal expansion.

The *E. hormaechei* strain (CG2039) in patient 24 and the *K. pneumoniae* ST967 strain (FK3112) from patient 20 harbored the same truncated plasmid (pFK3112-KPC-3), which may arise by recombination after the acquisition of intact plasmid. However, our conjugation experiment showed that pFK3112-KPC-3 cannot self-conjugate, and thus a possible explanation of the presence of pFK3112-KPC-3 in *E. hormaechei* and *K. pneumoniae* strain was that the same recombination happened independently in both species or pFK3112-KPC-3 was transferred with the assistance of helper plasmids. Our analysis demonstrated that plasmid-mediated horizontal and vertical transmission have played important roles in the KPC-3 Enterobacterales outbreak.

The *bla*_KPC-3_–harboring InX8 plasmid pCG2111-KPC-3 was almost identical to the *bla*_KPC-2_–harboring plasmids p13190–3 and p15WZ-82_KPC from *K. pneumoniae* and *K. variicola* strains in China, suggesting that KPC-3 probably originated through a single amino acid substitution on the same IncX8 plasmid. A similar KPC-2 to KPC-3 change has also been described in other *bla*_KPC_–harboring plasmids, including the epidemic pKpQIL-like plasmids (*25*). However, plasmids p13190–3 and p15WZ-82_KPC were identified in 7 (2013) and 5 (2015) years before the KPC-3 outbreak (2020), indicating that the *bla*_KPC-2_–harboring IncX8 plasmids have already existed and possibly circulated in China previously. 

Compared with the predominant *bla*_KPC-2_–harboring IncFII (pHN7A8)-R plasmids (>100 kb) in China, the *bla*_KPC-3_–harboring IncX8 plasmid has a smaller genome size (≈42 kb). Our results showed the conjugation frequencies of *bla*_KPC-3_–harboring IncX8 ranged from 1.57 × 10^−3^ to 7.8 × 10^−4^ per donor cell, which is similar to that of the *bla*_KPC-2_–harboring plasmids (6.3 × 10^−3^ to 1 × 10^−4^) (*26*–*28*) and the epidemic *bla*_NDM_–harboring IncX3 plasmids (*29*,*30*), which have spread widely across different sectors of the human population, the animal population, and the environment (*29*–*31*). Compared with the previously reported *bla*_KPC-2_–harboring plasmids (p13190-3 and p15WZ-82_KPC), our KPC-3 IncX8 plasmids have 1 less copy of iterons in the replication origin. The iterons are essential for plasmid replication and inhibition of plasmid overreplication (*32*). Whether the deletion of an iteron copy could affect the plasmid replication or copy numbers, leading to increase plasmid transfer, is unclear, which warrants further studies. Nevertheless, our study clearly demonstrates that IncX8 plasmids can transfer across different clinical Enterobacterales species. Our *G. mellonella* infection model results also indicated that the acquisition of *bla*_KPC-3_–harboring IncX8 in clinical hypervirulent ST65 K2 *K. pneumoniae* strains does not lead to reduced virulence. This observation could be another example of the emergence of carbapenem-resistant and hypervirulent *K. pneumoniae* strains attributable to the horizontal transfer of *bla*_KPC-3_–harboring IncX8 plasmids into prototypically hypervirulent K2 strains.

Although most KPC-3 producing strains were multidrug-resistant, most of them remained sensitive to ceftazidime/avibactam. In China, ceftazidime/avibactam has been approved for clinical treatment since 2019. However, resistance emerged soon after the clinical use of ceftazidime/avibactam in different regions, including China; this resistance usually was associated with mutations in the omega loop of KPC enzymes (*33*–*35*). Ceftazidime/avibactam resistance appeared to occur more frequently in a KPC-3 than a KPC-2 background, presumably because of the higher hydrolysis activity of KPC-3 against ceftazidime (*8*,*36*). Our subinhibitory concentrations ceftazidime/avibactam selection experiment results showed that most of our KPC-3–producing strains developed resistance, and the resistance rate was as high as 91.7%. By contrast, the KPC-2-producing strains showed a <4.2% rate for developing ceftazidime/avibactam resistance. However, PCR and Sanger sequencing of subinhibitory concentrations ceftazidime/avibactam–selected KPC-3 strains (3 colonies of each stain) failed to identify amino acid mutation in KPC-3 (data not shown). We suspected alternative mechanism, such as the increased gene copy numbers, expressions, or both (*37*,*38*), might contribute to ceftazidime/avibactam resistance. Nevertheless, our results suggested that the IncX8 *bla*_KPC-3_ strains may readily develop ceftazidime/avibactam resistance during treatment, despite being susceptible in vitro, which poses a major challenge for the clinical application of ceftazidime/avibactam as a last resort for treating CRE infections.

In this study, most patients had underlying diseases, had lengthy hospital stays, and underwent invasive medical device treatments (e.g., treatments involving a ventilator). Mechanical ventilation is a known risk factor of nosocomial infections, including CRE-attributable infections. The close proximity of these medical wards and movement of persons between the 2 buildings probably promoted the spread of KPC-3 strains between different wards. However, this outbreak went unrecognized and unconfirmed during routine surveillance, until our genomic study commenced in later 2021. Nevertheless, the COVID-19–related enhanced infection control measures already in place effectively controlled the KPC-3 outbreak. Unfortunately, this outbreak was not recognized and confirmed during routine surveillance, which should have detected the high numbers of multidrug-resistant *Serratia* infections as an unusual, and possibly epidemic, occurrence. Our results further emphasize that genomic surveillance and improved infection control practice are essential to tackle hospital outbreaks.

In summary, we report a KPC-3 *Enterobacterales* outbreak in China, which involved both clonal and horizontal transmissions of carbapenem resistance. The further spread of the *bla*_KPC-3_–harboring IncX8 plasmids and these KPC-3 strains in China and other global regions should be closely monitored.

Appendix 1 Materials and methods used for analyzing an outbreak of IncX8 plasmid–mediated KPC-3–producing *Enterobacterales* infection, China.

Appendix 2 Additional information about an outbreak of IncX8 plasmid–mediated KPC-3–producing *Enterobacterales* infection, China.
