# Supplementary material for: Outbreak of IncX8 Plasmid–Mediated KPC-3–Producing Enterobacterales Infection, China
Source: Emerg Infect Dis. 2022 Jul;28(7):1421–30. doi: 10.3201/eid2807.212181 (PMC9239885; doi:10.3201/eid2807.212181)
Supplement: Appendix 1 — Materials and methods used for analyzing an outbreak of IncX8 plasmid–mediated KPC-3–producing Enterobacterales infection, China. [file 21-2181-Techapp-s1.pdf]

Article DOI: https://doi.org/10.3201/eid2807.212181

Outbreak of IncX8 Plasmid–Mediated KPC-3–Producing *Enterobacterales* Infection, China

Appendix 1

Materials and Methods

Antimicrobial Susceptibility Testing and Modified Carbapenem Inactivation Method (mCIM)

The MICs (MICs) of 18 antimicrobial agents were determined by broth-microdilution assay (*1*). The results of antimicrobial susceptibility testing were interpreted under the Clinical and Laboratory Standards Institute (CLSI) guideline (*2*). The results for tigecycline and colistin were interpreted by European Committee on antimicrobial susceptibility testing (EUCAST) breakpoints (*3*). The *E. coli* ATCC25922 was used as quality control. The modified carbapenem inactivation method (mCIM) was performed to verify the carbapenemase production (*4*). The meropenem disks (10μg, OXOID, UK) were used for this study and *E. coli* ATCC25922 was acted as quality control bacteria for the mCIM assay.

WGS and Bioinformatics Analysis

Bacterial genomic DNA was isolated using the E.Z.N.A.® Bacteria DNA Kit (omega, USA). The DNA library was sequenced on the Illumina HiSeq platform by 150 bp paired-end reads. The raw data were filtered by Trimmomatic (*5*) v0.39, followed by assembly using Spades v3.15.2 (*6*). The Antimicrobial resistance genes, plasmid incompatibility groups, and multilocus sequence typing (MLST) were identified using the tools on Center for Genomic Epidemiology (CGE) web server (https://cge.cbs.dtu.dk/services/), including ResFinder 3.2, PlasmidFinder2.1,

and MLST 2.0, respectively. All the plasmids were extracted using the QIAGEN Plasmid Plus Maxi Kit according to the manual instruction. For plasmid sequencing, the plasmid DNA from the transconjugant or electro-transformant was subject to Illumina Hiseq sequencing, followed by de novo assembly using Spades v3.15.2n (*6*) as described above. Additional plasmids comparison was performed using Geneious prime 2020 software (https://www.geneious.com/). To determine the global phylogeny of *S. marcescens*, 748 genome assemblies were downloaded from the NCBI RefSeq database (dated as 10/01/2021), and these assemblies along with 18 genomes sequenced in this study were used to generate a core genome phylogenetic tree using Parsnp from Harvest suit (*7*) 1.12, followed by visualization and annotation using iTOL (*8*) v6. Core SNP distance was determined using the method described in our recent study (*9*). In brief, trimmed, paired-end sequences from each genome were mapped to *S. marcescens* 1140 genome (CP047688), using snippy (https://github.com/tseemann/snippy). Prophages were predicted using PHASTER (*10*), and repeated regions were examined using MUMmer 4.0 (*11*) while regions of recombination were detected using Gubbins v3.1.0 (*12*) Single nucleotide polymorphisms (SNP) among prophages, repeated or recombination regions were filtered, and the core SNP differences were determined using SNP-dists (https://github.com/tseemann/snp-dists). Bacterial species and potential contamination were examined by Mash 2.2 using the NCBI RefSeq genome (*13*). *Enterobacter* subspecies were determined using fast ANI v1.33 (*14*) against the type strain genomes as described in a recent study of precise species identification for *Enterobacter* (*15*).

Conjugation and Electroporation Experiments

The conjugation experiments were performed using *E. coli* EC600 (rifampin-resistant) as the recipient strain and 8 selected $bla_{\text{KPC-3}}$-harboring clinical isolates as the donors, using the method described previously (*4*,*16*). All transconjugants were subjected to PCR detection for the presence of $bla_{\text{KPC-3}}$ gene, followed by susceptibility testing to examine the resistant phenotypes. The conjugation frequency was determined by dividing the numbers of transconjugants by the

number of donors. If transconjugants weren't obtained, electroporation was conducted to obtain the electro-transformants, using the method described previously (*17*).

Pulsed-Field Gel Electrophoresis(PFGE) and S1-PFGE

The Pulsed-field gel electrophoresis (PFGE) was performed to explore the clonal relationship among 8 selected CRE isolates, while S1-PFGE was used to determine the plasmid profile of bla_{KPC-3} -harboring strains and their transconjugants, using the previously described methods (*18*). The *XbaI* digested *Salmonella* Braenderup H9812 strain was used as the molecular size marker.

String Test and *Galleria Mellonella* Infection Model

Hypermucoviscous phenotypes of *K. pneumoniae* strains were examined by string test and a viscous string longer than 5 mm was defined as positive. The virulence potential of ST65 K2 *K. pneumoniae* strains was evaluated using a *Galleria Mellonella* larvae infection model. The larvae were obtained from HuiYuDe Biotech Co, Tianjin, China, and larva weighing 250–350 mg were used. Two KPC-3-producing ST65 K2 *K. pneumoniae* isolates (FK3015 and FK3018) were tested. An ST439 K2 hypervirulent reference strain ATCC 43816 was used as the positive control (*19*). PBS injected and uninfected (injected an "empty syringe") groups were used as negative controls. In addition, one clinical multi-drug resistant *K. pneumoniae* strain isolated from the same hospital was included for comparison. Ten larvae were randomly divided into each group. The *K. pneumoniae* groups were injected with 10μl (~1.5×10^7 CFU) bacterial suspension into the haemocoel of each larva via the last left proleg, while the negative control groups were injected with PBS or empty syringe. Larvae were grown in petri dishes at 37°C, and survival was observed and scored every hour. All experiments were performed in triplicates. The result was analyzed and visualized using Prism 8.0.2 software (GraphPad, USA).

Ceftazidime/avibactam in *vitro* Selection Assay

Previous studies showed KPC-3 had ~30 fold higher ceftazidime catalytic activity in comparison to KPC-2, and KPC-3 was easily selected to be resistant to ceftazidime/avibactam (*17*,*20*). In *vitro* selection assay was conducted in all KPC-3-producing strains (except the NDM-1 and KPC-3 co-producing *E. hormaechei* strain CG2126) to examine the potential of inducted ceftazidime/avibactam resistance. Single clone of the tested strain was added into 5ml Luria Bertani (LB) medium containing $^{1}/_{2}$ MIC concentration of ceftazidime/avibactam, and incubated overnight at 37°C, 220rpm/min. Then bacteria were collected by centrifugation (12000-rpm, 1 min), followed by washing three times with phosphate buffered saline (PBS). The bacterial was then diluted to 10^7 CFU and used to measure the MIC values using the broth micro dilution method according to CLSI 2020 (*2*), and the experiment was repeated three times.

References

1. Clinical and Laboratory Standards Institute. Performance standards for antimicrobial susceptibility testing. Twenty-second informational supplement. Wayne (PA): The Institute; 2012.

2. Clinical and Laboratory Standards Institute. Performance Standards for Antimicrobial Susceptibility Testing. CLSI supplement M100, 30th edition. Wayne (PA): The Institute; 2020.

3. European Committee on Antimicrobial Susceptibility Testing. Breakpoint tables for interpretation of MICs and zone diameters. Version 10.0 [cited 2020 Jan 20]. http://www.eucast.org

4. Shen X, Liu L, Yu J, Cao X, Zhan Q, Guo Y, et al. Coexistence of *bla*$_{\text{NDM-1}}$ and *rmtC* on a transferrable plasmid of a novel ST192 clinical isolate. Infect Drug Resist. 2019;12:3883–91. PubMed https://doi.org/10.2147/IDR.S228130

5. Bolger AM, Lohse M, Usadel B. Trimmomatic: a flexible trimmer for Illumina sequence data. Bioinformatics. 2014;30:2114–20. PubMed https://doi.org/10.1093/bioinformatics/btu170

6. Bankevich A, Nurk S, Antipov D, Gurevich AA, Dvorkin M, Kulikov AS, et al. SPAdes: a new genome assembly algorithm and its applications to single-cell sequencing. J Comput Biol. 2012;19:455–77. PubMed https://doi.org/10.1089/cmb.2012.0021

7. Treangen TJ, Ondov BD, Koren S, Phillippy AM. The Harvest suite for rapid core-genome alignment and visualization of thousands of intraspecific microbial genomes. Genome Biol. 2014;15:524. PubMed https://doi.org/10.1186/s13059-014-0524-x

8. Letunic I, Bork P. Interactive Tree Of Life (iTOL) v5: an online tool for phylogenetic tree display and annotation. Nucleic Acids Res. 2021;49(W1):W293–6. PubMed https://doi.org/10.1093/nar/gkab301

9. Yu F, Cienfuegos-Gallet AV, Cunningham MH, Jin Y, Wang B, Kreiswirth BN, et al. Molecular evolution and adaptation of livestock-associated methicillin-resistant *Staphylococcus aureus* (LA-MRSA) sequence type 9. mSystems. 2021;6:e0049221. PubMed https://doi.org/10.1128/mSystems.00492-21

10. Arndt D, Grant JR, Marcu A, Sajed T, Pon A, Liang Y, et al. PHASTER: a better, faster version of the PHAST phage search tool. Nucleic Acids Res. 2016;44(W1):W16-21. PubMed https://doi.org/10.1093/nar/gkw387

11. Kurtz S, Phillippy A, Delcher AL, Smoot M, Shumway M, Antonescu C, et al. Versatile and open software for comparing large genomes. Genome Biol. 2004;5:R12. PubMed https://doi.org/10.1186/gb-2004-5-2-r12

12. Croucher NJ, Page AJ, Connor TR, Delaney AJ, Keane JA, Bentley SD, et al. Rapid phylogenetic analysis of large samples of recombinant bacterial whole genome sequences using Gubbins. Nucleic Acids Res. 2015;43:e15. PubMed https://doi.org/10.1093/nar/gku1196

13. Ondov BD, Treangen TJ, Melsted P, Mallonee AB, Bergman NH, Koren S, et al. Mash: fast genome and metagenome distance estimation using MinHash. Genome Biol. 2016;17:132. PubMed https://doi.org/10.1186/s13059-016-0997-x

14. Jain C, Rodriguez-R LM, Phillippy AM, Konstantinidis KT, Aluru S. High throughput ANI analysis of 90K prokaryotic genomes reveals clear species boundaries. Nat Commun. 2018;9:5114. PubMed https://doi.org/10.1038/s41467-018-07641-9

15. Wu W, Feng Y, Zong Z. Precise species identification for *Enterobacter*: a genome sequence-based study with reporting of two novel species, *Enterobacter quasiroggenkampii* sp. nov. and *Enterobacter quasimori* sp. nov. mSystems. 2020;5:e00527–20. PubMed https://doi.org/10.1128/mSystems.00527-20

16. Papagiannitsis CC, Bitar I, Malli E, Tsilipounidaki K, Hrabak J, Petinaki E. IncC bla_{KPC-2}-positive plasmid characterised from ST648 *Escherichia coli*. J Glob Antimicrob Resist. 2019;19:73–7. PubMed https://doi.org/10.1016/j.jgar.2019.05.001

17. Shields RK, Chen L, Cheng S, Chavda KD, Press EG, Snyder A, et al. Emergence of ceftazidime-avibactam resistance due to plasmid-borne bla_{KPC-3} mutations during treatment of carbapenem-resistant *Klebsiella pneumoniae* infections. Antimicrob Agents Chemother. 2017;61:e02097–16. PubMed

18. Noormohamed A, Fakhr MK. Molecular typing of and isolated from various retail meats by MLST and PFGE. Foods. 2014;3:82–93. PubMed https://doi.org/10.3390/foods3010082

19. Fodah RA, Scott JB, Tam H-H, Yan P, Pfeffer TL, Bundschuh R, et al. Correlation of *Klebsiella pneumoniae* comparative genetic analyses with virulence profiles in a murine respiratory disease model. PLoS One. 2014;9:e107394. PubMed https://doi.org/10.1371/journal.pone.0107394

20. Haidar G, Clancy CJ, Shields RK, Hao B, Cheng S, Nguyen MH. Mutations in bla_{KPC-3} that confer ceftazidime-avibactam resistance encode novel KPC-3 variants that function as extended-spectrum β-lactamases. Antimicrob Agents Chemother. 2017;61:e02534–16. PubMed https://doi.org/10.1128/AAC.02534-16
